# Habits of a highly successful cereal killer, *Striga*

**DOI:** 10.1371/journal.ppat.1006731

**Published:** 2018-01-11

**Authors:** Steven Runo, Eric K. Kuria

**Affiliations:** Department of Biochemistry and Biotechnology, Kenyatta University, Nairobi, Kenya; THE SAINSBURY LABORATORY, UNITED KINGDOM

## Introduction

*Striga* is a highly successful pathogen of cereal crops in sub-Saharan Africa. Also known as witchweed, *Striga* is an attractive parasitic plant whose beautiful flowers belie its noxiousness. Most cultivated cereals, including maize, millet, sorghum, and rice, are parasitized by at least one *Striga* species, leading to enormous economic losses. Control strategies are limited but include common agronomic practices of hand weeding, crop rotation, and general sanitization techniques. *Striga*-resistant crops, as well as tolerant ones, have also been used, but this resistance tends to break down with the emergence of new *Striga* variants. With limited and ineffective management options, *Striga* has continued to increase both its host range and area under infestation. In this article, we outline seven unique characteristics of *Striga* as a parasite of great economic importance, explore reasons for its success, and outline emerging control options.

## 1. Diversity

**Fig 1:**
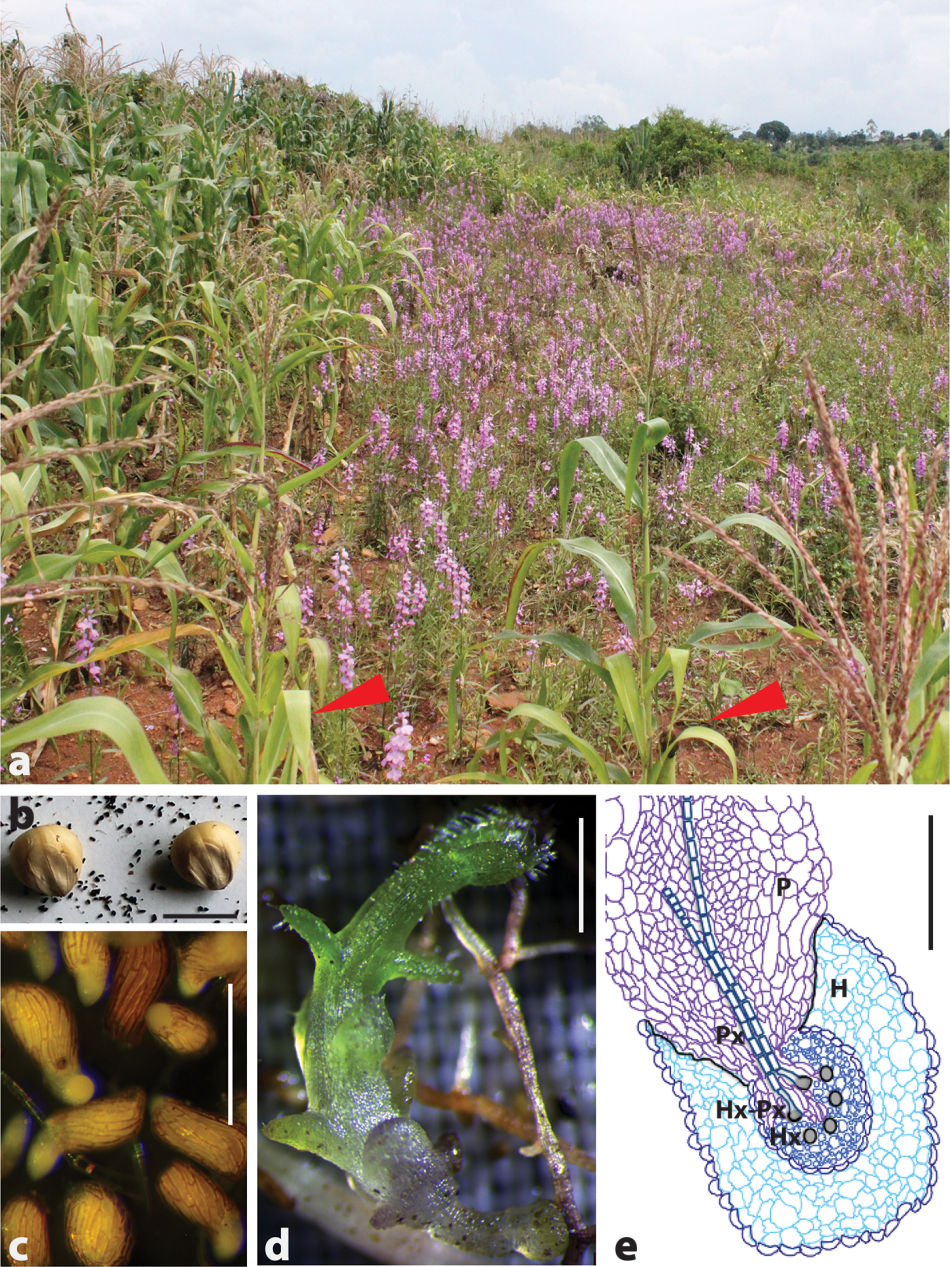
Witchweed (*Striga*) at a glance. (a) Destructive effect of *S*. *hermonthica* at high infestation (right), where *Striga* has caused complete crop failure, compared with low infestation (left), where some yields can be realized. Other effects of *Striga* infestation such as chlorosis can be observed on the field in the right (red arrows). This picture was taken in a farmer’s maize field in Gem, Siaya County in Western Kenya. Photo credit Daniel Ajaku (ICRISAT). (b) *Striga* small seeds in comparison with sorghum. The tiny dust-like seeds can remain dormant in soil for decades. Scale bar = 5 mm. (c) *Striga* seedlings 12 hours after treating with a germination stimulant (GR24). *Striga* seeds germinate only in response to chemical cues from the host plant. Scale bar = 0.5 mm. (d) A 10-day–old *Striga* seedling with a well-developed haustorium attached to a sorghum root. Scale bar = 0.2 mm. (e) A schematic of a section through a 10-day–old *Striga* seedling showing the parasite (P) attached to a host (H). By this time, the parasite has developed fully. The parasite xylem (Px) has merged with the host xylem (Hx) to form a siphon (Hx-Px) that sucks out nutrients from the host. Scale bar = 0.05 mm. H, host; Hx, host xylem; ICRISAT, International Crops Research Institute for the Semi-Arid Tropics; P, parasite; Px, parasite xylem.

The *Striga* genus has many species that are widely distributed, each with different host preferences. While some *Striga* species can only infect one or a few host species, others can infect many host genera. The diversity in host preference by members of the *Striga* genus ensures ease of finding suitable hosts, which helps expand its range. Consequently, *Striga* has been able to expand from its native range in the Semien hills of Ethiopia and the Nubian Hills of Sudan to over 40 countries in Africa [1]. In infested fields, *Striga* causes 20% to 100% crop losses as illustrated in Fig 1A. Such crop losses lead to great economic losses. For example, *Striga* causes an estimated loss of $111 million to $200 million annually in rice alone [2].

With regard to specific species distribution and host range, *S*. *hermonthica*’s range extends throughout Eastern Africa in Kenya, Uganda, Tanzania, Ethiopia, Sudan, Rwanda, and Burundi as well as in West Africa, where it parasitizes maize, millet, sorghum, and upland rice. *S*. *asiatica* occurs in both Southern and Eastern Africa, the Arabian Peninsula, India, and Pakistan and is parasitic to rice, sorghum, and millet. *S*. *gesnerioides* infects dicotyledonous plants, greatly limiting cowpea production in the West African countries of Mali, Burkina Faso, Niger, and Benin. Finally, *S*. *aspera* constrains maize and rice production in Sudan, Malawi, Nigeria, Cameroon, Ivory Coast, and Senegal [3].

## 2. Fecundity

Fruitfulness and fertility in biology are used to describe an organism that is able to produce a large number of viable offspring. A single *Striga* plant produces 50,000 to 500,000 seeds [4]. Such high fecundity provides *Striga* with high epidemiological potential because of the exponential increase in pathogen population from one generation to the next.

## 3. Mobility

*Striga* seeds are small (about 0.2 mm in length) and dust-like (Fig 1B). *Striga*’s small seed size greatly enhances the parasite’s dispersal by wind, water, and contaminated crop seeds. The small-sized seeds also adhere to people and animals, further aiding in seed spread and contamination of uninfected fields. However, a small *Striga* seed size means that the endosperm can only support growth for three to seven days [4]. Therefore, germination must be rapid and synchronized with host localization.

## 4. Coordinated

The *Striga* parasitic life cycle is highly synchronized with its host and involves the following three general phases: germination, haustorium formation, and penetration (Fig 1C, 1D and 1E). Germination only occurs in the presence of a suitable host, which *Striga* locates using plant hormones called strigolactones. Physiologically, strigolactones function to inhibit shoot branching in plants [5] but also serve as cues for colonization by phosphate (P)-mobilizing symbionts such as arbuscular mycorrhiza fungi (AMF) [6]. Accordingly, when P levels in soil fall, plant roots exude strigolactone to increase mycorrhization by AMF. However, *Striga* “eavesdrops” on this conversation and hijacks the signal [6]. Strigolactone then binds to receptors—*KARRAKIN-INSENSITIVE 2 (KAI2)/HYPERSENSITIVE TO LIGHT (HTL)* [7]—in *Striga* seeds, causing the receptor to recruit an F-box protein that degrades germination-inhibiting transcriptional regulators. These series of events trigger *Striga* seed germination [8].

Following germination, the *Striga* radicle attaches to the host and differentiates into a specialized organ—the haustorium (Fig 1D and 1E) [9]. Like in germination, the haustorium develops in response to chemical cues from the host, the haustorial inducing factors such as 2,6-dimethoxy-p-benzoquinone (DMBQ) [10].

Finally, the haustorium penetrates the host until it encounters the endodermis, where haustorial cells elongate and divide to establish vascular connections with the host (Fig 1D and 1E). Once the vascular systems of the host and parasite are connected, *Striga* uses this connection to siphon out nutrients.

## 5. Dormancy

In the absence of the germination signal, *Striga* exhibit high dormancy and longevity. *Striga* seeds can remain dormant in soil for up to 10 years, waiting for the appropriate host to attack [11]. The parasite’s ability to “cool off” for extended periods of time is important in its life cycle because it renders the parasite resistant to herbicides as well as decay-inducing organisms.

## 6. Manipulative

*Striga* reprograms the host in ways that are beneficial to its parasitism. It has been shown that (i) *Striga* is able to alter levels of important hormone such as abscisic acid (ABA), cytokinins, and gibberellic acid during infection [12] and (ii) acquire genes from its host and modify them for parasite use [13].

Furthermore, it is now emerging that *Striga* is a sophisticated manipulator of host immunity [14]. Plausibly, *Striga* is able to subdue host defense by producing a battery of molecules (effectors), just like bacterial and fungal pathogens. Although identification and characterization of effector genes from *Striga* has not been carried out, race-specific activation of host defense response lends credence to this hypothesis. It has been shown that in a *S*. *gesnerioides*–resistant cowpea variety (b301), hypersensitive reaction (HR) immunity is only activated by the hypervirulent *S*. *gesnerioides* race 3 (SG3) from Nigeria and Niger [15].

The *Striga* effector paradigm is more plausible when one draws extrapolations from other parasitic plant–host interactions. For example, potential effectors have been recently described in *Orobanche ramosa*–*Arabidopsis* [16] and in the *Cuscuta reflexa*–tomato interactions [17].

## 7. Mysterious

*Striga*’s common name, “witchweed,” is derived from the mysterious drought-like symptoms, wilting, and chlorosis seen in *Striga*-infected plants even before the parasite emerges from soil (Fig 1A). This effect occurs very soon after infection—as early as one week after infection—and cannot be attributed to simply *Striga* sucking out nutrients from the host. It has been long suggested that *Striga* injects yet-to-be-identified phytotoxins to its hosts during parasitism, causing the “bewitching” phenotype [18]. Although research on detailed characterization of the toxin and its mechanisms of action remains elusive, support for the hypothesis that *Striga* contains powerful secondary metabolites that help the parasite subdue its hosts can be derived from phytochemical studies of *Striga* extracts, for example iridoid glucosides [19]. Although not toxic themselves, iridoic glycosides are easily converted to toxic aglycone moiety [20], which poisons cells by denaturing proteins.

## Summary and perspectives

Characteristics of *Striga* described above are indicative of a successful pathogen whose control should take into account biological and chemical mechanisms underpinning *Striga*–host interaction. Therefore, common emerging *Striga* control strategies are aimed at (i) exploiting host-based resistance, (ii) evading *Striga* germination, and (iii) depleting the vast *Striga* seedbank in soil.

### Exploiting host-based resistance

Some crops and their wild relatives are able to block *Striga* penetration through biochemical [21] or mechanical [22] barriers. In addition, some *Striga* hosts resist parasitism by triggering an HR at the site of infection [15]. Current advances in genomics and molecular genetics provide tools for “pinpointing” resistance genes and/or loci through genome-wide association mapping and transcriptome profiling. Identified genes and/or loci can then be combined (pyramided) to provide durable resistance.

### Evading *Striga* germination

Some crop varieties produce variants of strigolactone that do no induce *Striga* germination effectively. In sorghum, this was found to be due to a mutation on the *LOW GERMINATION STIMULANT 1 (LGS1)* locus [23]. This development provides prospects for manipulating the strigolactone production pathway in crops—using genome-editing tools—in order to develop crop varieties less potent in inducing *Striga* germination. *Striga* germination can also be blocked by small molecules that bind to the strigolactone receptor in *Striga* as antagonist [24], pointing to the possibility of using chemical biology technology in *Striga* control.

### Depleting *Striga* seedbank

Chemical molecules that mimic the activity of strigolactones present a new frontier in *Striga* control because such molecules can cause the parasite to germinate in the absence of a host—a strategy known as suicidal germination [25]. This technology was previously not feasible due to the high cost associated with multistep chemical synthesis of strigolactones as well as their instability under field conditions. It is now possible to synthesize simple strigolactone analogues that, when treated with chemical formulations, retain activity and stability under field conditions [25]. This approach is attractive because in the short term, it can lead to a significant reduction of *Striga* seeds in soil.

In summary, while advances in science provide additional tools for *Striga* control, these measures should be employed in combination with more traditional approaches such as crop rotation and sanitizing to minimize *Striga* seed increase and spread to noncontaminated fields.
